# Reconstructing historical catch trends of threatened sharks and rays based on fisher ecological knowledge

**DOI:** 10.1111/cobi.70059

**Published:** 2025-05-31

**Authors:** Guido Leurs, Rima W. Jabado, Assana Camará, Lilísio Dos Santos, Diosnes Manuel Nonque, Thije J. Zuidewind, Iça Barry, Pierre Campredon, Benja Blaschke, Karin de Boer, Nadia Hijner, Han Olff, Samuel Ledo Pontes, Aissa Regalla, Matthew Bjerregaard Walsh, Laura L. Govers

**Affiliations:** ^1^ Conservation Ecology Group, Groningen Institute for Evolutionary Life Sciences University of Groningen Groningen The Netherlands; ^2^ Marine Animal Ecology Group Wageningen University & Research Wageningen The Netherlands; ^3^ Elasmo Project Dubai UAE; ^4^ College of Science and Engineering James Cook University Townsville Australia; ^5^ Instituto Nacional de Investigação das Pescas e Oceanografia (INIPO) Bissau Guinea‐Bissau; ^6^ Marine and Environmental Sciences Centre (MARE)/Aquatic Research Network (ARNET) ISPA Instituto Universitário Lisbon Portugal; ^7^ ISCTE University Institute of Lisbon Lisbon Portugal; ^8^ University of the Azores Ponta Delgada Portugal; ^9^ Instituto da Biodiversidade e das Áreas Protegidas (IBAP) Bissau Guinea‐Bissau; ^10^ Beta Science Shop, Faculty of Science and Engineering University of Groningen Groningen The Netherlands; ^11^ Food and Agriculture Organization of the United Nations (FAO) Rome Italy; ^12^ Department of Coastal Systems, Royal Netherlands Institute for Sea Research (NIOZ) Den Burg The Netherlands

**Keywords:** coastal ecology, conservation, conservation ecology, elasmobranchs, fisheries, Guinea‐Bissau, local ecological knowledge, small‐scale fisheries, West Africa, África Occidental, conocimiento ecológico local, conservación, ecología costera, ecología de la conservación, elasmobranquios, Guinea‐Bisáu, pesquerías, pesquerías artesanales

## Abstract

Small‐scale fisheries often lack historical shark and ray catch information, hampering their management. We reconstructed historical catch trends and current fishing pressure by combining local ecological knowledge, satellite‐based vessel counts, and a short‐term landing‐site survey. To test the effectiveness of this method, we focused on the Bijagós Archipelago (Guinea‐Bissau, West Africa), where historical fisheries data are lacking. Benthic rays (stingrays [Dasyatidae] and butterfly rays [*Gymnura* spp.]), benthopelagic rays (duckbill eagle rays [*Aetomylaeus bovinus*] and cownose rays [*Rhinoptera marginata*]), guitarfish (*Glaucostegus* and *Rhinobatos* spp.), requiem sharks (Carcharhinidae), and hammerhead sharks (*Sphyrna* spp.) declined in abundance by 81.5–96.7% (species dependent) from 1960 to 2020. Fishing effort increased annually: fishing trip duration by 42.0% (SE 3.4), numbers of fishing vessels at sea as perceived by fishers by 36.3% (1.0) (1960–2020), and number of vessels by 12.0% (1.1) (2007–2022). We estimated that in 2020, fishing vessels collectively captured 61–264 sharks and 522–2194 rays per day in the archipelago, depending on the proportion of the fishing fleet that was active (i.e., low fleet activity of 18% and high fleet activity of 80%). We advocate for reducing shark and ray catches by regulating fleet size, reinforcing boundaries of protected areas, and collecting fisher‐dependent information on shark and ray landings to safeguard these vulnerable species and coastal livelihoods. We demonstrated the effectiveness of using this 3‐pronged approach to provide baseline data on shark fisheries, a common challenge in areas with small‐scale fisheries and limited research capacity.

## Abstract (es)

Reconstrucción histórica de las tendencias de captura de tiburones y rayas amenazadas con base en el conocimiento ecológico de los pescadores

**Resumen:**

Las pesquerías artesanales suelen carecer de información histórica sobre las capturas de tiburones y rayas, lo que dificulta su gestión. Reconstruimos las tendencias históricas de las capturas y la presión pesquera actual combinando los conocimientos ecológicos locales, el recuento de embarcaciones por satélite y un estudio a corto plazo de los lugares de desembarque. Para probar la eficacia de este método, nos centramos en el archipiélago de Bijagós (Guinea‐Bisáu, África Occidental), donde faltan datos históricos de pesca. Trabajamos con rayas bentónicas (rayas látigo (Dasyatidae)) y rayas mariposa (*Gymnura* spp.), rayas bentopelágicas (rayas águila pico de pato (*Aetomylaeus bovinus*) y rayas nariz de vaca (*Rhinoptera marginata*)), peces guitarra (*Glaucostegus* y *Rhinobatos* spp.), los tiburones réquiem (Carcharhinidae) y los tiburones martillo (*Sphyrna* spp.) disminuyeron su abundancia entre un 81.5 y un 96.7% (según la especie) entre 1960 y 2020. El esfuerzo pesquero aumentó anualmente: la duración de las mareas en un 42% (ES 3.4), el número de buques pesqueros en el mar percibido por los pescadores en un 36.3% (1.0) (1960‐2020) y el número de buques en un 12% (1.1) (2007‐2022). Estimamos que, en 2020, los buques pesqueros capturaron colectivamente entre 61 y 264 tiburones y entre 522 y 2,194 rayas al día en el archipiélago, dependiendo de la proporción de la flota pesquera activa (es decir, una actividad baja de la flota del 18% y una actividad alta de la flota del 80%). Abogamos por reducir las capturas de tiburones y rayas regulando el tamaño de la flota, reforzando los límites de las zonas protegidas y recopilando información dependiente de los pescadores sobre los desembarcos de tiburones y rayas para salvaguardar estas especies vulnerables y los medios de subsistencia costeros. Demostramos la eficacia de utilizar este triple enfoque para proporcionar datos de referencia sobre la pesca de tiburones, un reto habitual en zonas con pesquerías artesanales y una capacidad de investigación limitada.

## INTRODUCTION

The impact of global fisheries on marine ecosystems, marine biodiversity, and fish populations is profound (Jackson et al., 2001; Lotze, 2007). These changes have been linked to shifts in ecosystem functioning and a loss of ecosystem services (Jackson et al., 2001; Lotze et al., 2006; Worm et al., 2006). One of the most affected species groups is sharks and rays (i.e., elasmobranchs), evidenced by their deteriorating conservation status (i.e., Stevens et al. [2000] and Dulvy et al. [2021]). An estimated one third of all elasmobranch species is threatened with extinction (Dulvy et al., 2021). The impact of industrial fisheries on shark and ray populations has been documented extensively (e.g., Baum et al., 2003; Queiroz et al., 2019; Worm et al., 2013). Many of these fisheries are managed through regional fisheries bodies and fishing agreements (e.g., sustainable fisheries partnership agreements), which include requirements on catch data reporting and vessel monitoring systems. This vessel monitoring information can be used to trace industrial fisheries and predict potential illegal activities (Kroodsma et al., 2018; Welch et al., 2022). In comparison, tracking and managing small‐scale fisheries (SSF) may be more complicated, and their impact on shark and ray populations often remains undocumented. In coastal areas, where most shark and ray species occur, the combined effects of fisheries and habitat degradation are disproportionately high (Dulvy et al., 2021). Here, elasmobranchs are affected mainly by SSF (i.e., defined as fisheries involving small vessels of up to 12 m in length and typically minimal use of technological gear [Chuenpagdee et al., 2006; Guillemot et al., 2014]), and their interaction with industrial fisheries is limited. Globally, SSF catches make up a large proportion of total fish catches (Béné et al., 2015; Palomares & Pauly, 2019; Teh & Pauly, 2018), especially where these fisheries are linked closely with local communities and are essential for food security (Béné, 2006; Palomares & Pauly, 2019). Small‐scale fisheries have increased steadily over the past decades (Palomares & Pauly, 2019; Teh & Pauly, 2018) and can have highly targeted or incidental or both catches of elasmobranchs (e.g., Haque et al., 2021a; Karnad et al., 2020; Temple et al., 2019).

Due to the spatially concentrated nature of SSF in nearshore areas (e.g., Metcalfe et al., 2017), their overlap with coastal shark and ray species can be relatively high, likely exerting high localized pressure on their populations. In addition, these fisheries can affect the vulnerable early life stages of elasmobranch species using nearshore areas as nursery or feeding areas (Doherty et al., 2023; Humber et al., 2017; Knip et al., 2010). Despite the increase of these fisheries and their importance to local communities for income or subsistence (Haque et al., 2021b; Teh & Pauly, 2018), they often remain unregulated with little reporting of catches (Agyeman et al., 2021; Belhabib & Pauly, 2015; Belhabib et al., 2014; Ekpo & Essien‐Ibok, 2019; Haque et al., 2021a). Such limited data availability and low traceability of fishing effort make the assessment of the impact of these fisheries on sharks and rays challenging.

We sought to determine the historical and current population trends of sharks and rays where fisheries‐dependent data collection is limited or nonexistent. We focused on one of the largest coastal ecosystems in one of the most data‐deficient regions of the world: the Bijagós Archipelago off the coast of Guinea‐Bissau in West Africa. In West Africa, industrial fisheries and SSF have expanded rapidly since the 1990s (Campredon & Cuq, 2001; Lemrabott et al., 2023; Leurs et al., 2021). Sharks and rays are often targeted or retained when incidentally caught because their fins are sold in international markets and their meat is sold in local or regional markets (Dia et al., 2023; Diop & Dossa, 2011). Coastal areas in the region are potentially important for the various life‐history stages of sharks and rays (Campredon & Cuq, 2001; Knip et al., 2010; Leurs, Nieuwenhuis, et al., 2023a; Leurs, Verkuil, et al., 2023b; Valadou et al., 2006). However, it is unclear how SSF have affected these species, what their current status is, and how high the current fishing pressure is. To determine the historical and current catches and likely population trends of sharks and rays in the Bijagós Archipelago, we used a novel 3‐pronged approach. We combined fisher local ecological knowledge (LEK), satellite‐based small‐scale fishing vessel counts, and a short‐term landing‐site survey. Specifically, we estimated the historical catch trends of shark and ray species based on fisher LEK, evaluated changes in fishing effort based on the number of fishing vessels, fishing trip duration, and gear use, and estimated the daily catches of sharks and rays under different levels of small‐scale vessel activity scenarios.

## METHODS

### Study area

**FIGURE 1:**
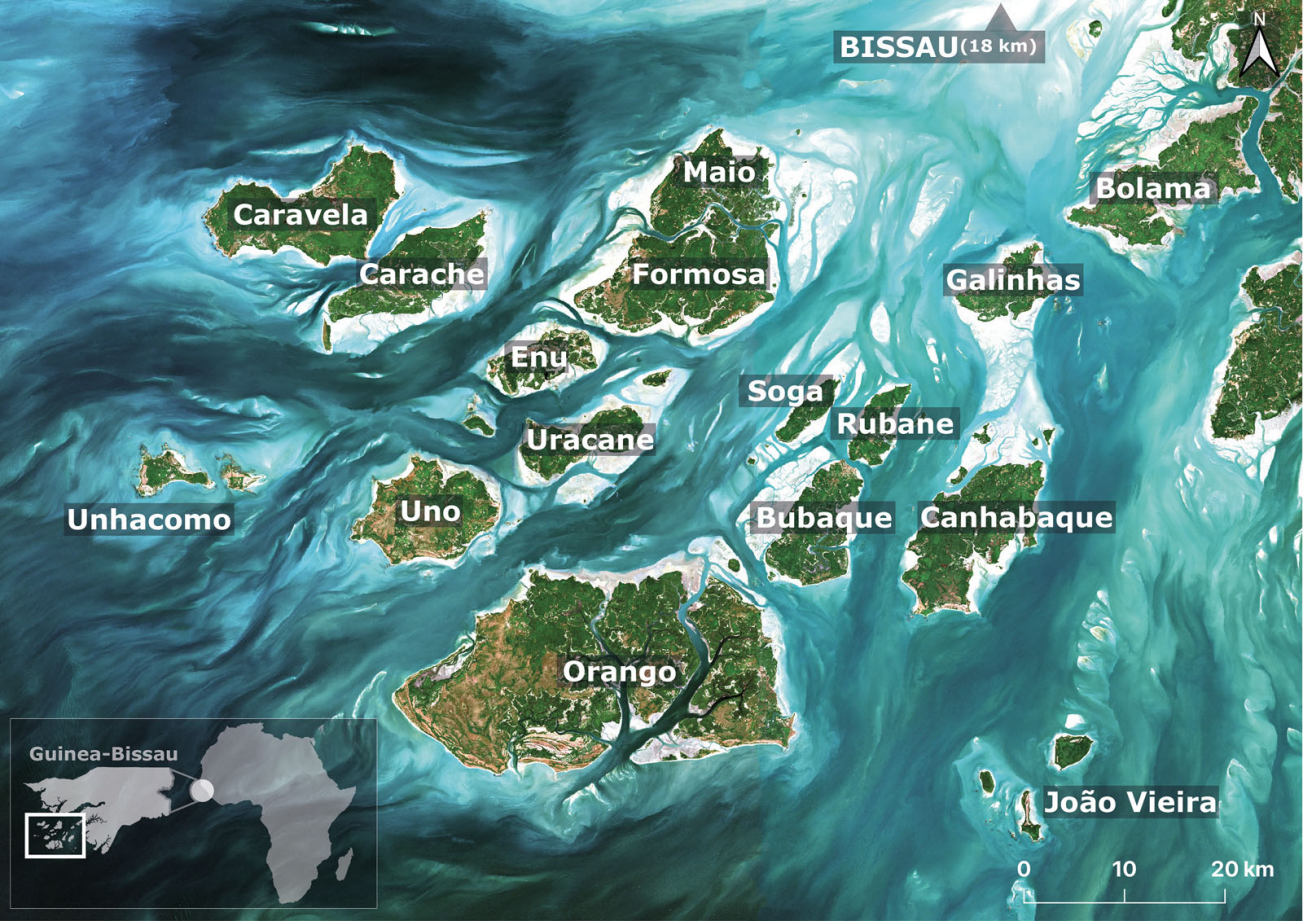
The Bijagós Archipelago (11°15ʹN, 16°05ʹW) in Guinea‐Bissau.

The Bijagós Archipelago (11°15ʹN, 16°05ʹW) off Guinea‐Bissau consists of 88 islands, about 20 of which are permanently inhabited (Figure 1). Situated in the Geba River estuary, it features mangrove‐fringed islands, intertidal flats, and tidal creeks. The archipelago is a UNESCO Biosphere Reserve since 1996 and a Ramsar site since 2014; its sharks and rays have cultural significance for its people (Cross, 2014); and it is a large intertidal ecosystem (Leurs et al., 2023b). Traditionally used in ceremonies, these species face increasing pressure from international demand, driving targeted and incidental fisheries across West Africa (Campredon & Cuq, 2001; Diop & Dossa, 2011). Since 1985, SSF in the archipelago have expanded from seasonal to year‐round operations, often targeting sharks and rays (Campredon & Cuq, 2001). Fishers use dugout canoes, wooden pirogues with outboard engines, or beach seines (Appendix S1). Historical and current artisanal fishery catch data are lacking. The archipelago has 3 marine protected areas (MPAs): João Vieira‐Poilão Marine Park, Orango National Park, and the community‐managed Urok MPA (Figure 1). Although regulations prohibit monofilament nets and shark and ray targeting (Diop & Dossa, 2011), enforcement remains weak due to limited capacity.

### Elicitation of fishers’ ecological knowledge

Two local researchers were trained to conduct in‐depth semistructured interviews with fishers who operate in the Archipelago. Interviews were conducted in Portuguese Creole from February to June 2021 at the largest small‐scale fish market in the country: the Alto Bandim fish market in Bissau (11°50ʹ29ʺ N, 15°35ʹ19ʺ W). Snowball sampling was used in that respondents were asked to recommend other fishers to interview (Goodman, 1961), although fishers were selected to ensure even sampling across age groups present in the fishing community. This meant that specifically younger or older fishers were selected based on snowball sampling to ensure that the entire age range of the Bijagó fishing community was represented in the final data set.

To increase the accuracy of data collected from fishers and examine change in variables over time, we asked about the significant dates a fisher could recall best: when the fisher started fishing and the most recent year a fisher had fished. If a fisher was not active anymore, we asked what was the last year in which they fished (e.g., Tesfamichael et al., 2014). Although this approach leads to fewer data points per fisher, the information collected was likely that which fishers could recall most accurately (Appendix S3). This approach of capturing temporal changes was used for all variables in the interview.

From the interviews, we determined changes in shark capture and fishing gear (e.g., we asked fishers about the duration of fishing trips and catch composition now and when they started fishing). This method yields results comparable to those based on catch and effort data but based on the recollection of fishers (Tesfamichael et al., 2014). The central objective of the interviews was to capture perceptions of changes in species abundance, fishing effort, gear use, and species between when a fisher started fishing and in the last study year when the interview took place. Fishers were asked for demographic information and about fishing gear use, areas fished, and species‐specific captures. Open‐ended questions focused on the current fisheries management and other information fishers wanted to share (Appendix S2). Photographic species cards were used to establish a mutual understanding of species identity. Because species in Creole are grouped and fishers were unable to differentiate between species, we grouped species in the following functional groups: benthic rays (stingrays [*Hypanus* spp., *Dasyatis* spp., *Fontitrygon* spp.] and butterfly rays [*Gymnura* spp.]), benthopelagic rays (duckbill eagle ray [*Aetomylaeus bovinus*] and Lusitanian cownose ray [*Rhinoptera marginata*]), guitarfishes (common guitarfish [*Rhinobatos rhinobatos*] and blackchin guitarfish [*Glaucostegus cemiculus*]), requiem sharks (*Carcharhinus* spp. and milk shark [*Rhizoprionodon acutus*]), and hammerhead sharks (*Sphyrna* spp.) (Appendix S4). For each group, specific information, such as individuals caught per fishing expedition, average length of captured individuals, processing, and trade, was recorded. Fishers were asked to indicate the total lengths (or disc widths for stingrays) of an average individual on a representative fishing trip in the year the fisher started fishing and at the time of the interview (or when the fisher stopped fishing). The enumerators documented these estimates by comparing the indicated sizes with a measuring tape. Interviews lasted from 1 to 2.5 h because fishers were allowed to expand on their experience.

### Ethics statement

Before each interview, the purpose of the interview and the study's objectives were explained to each potential participant, and formal consent was obtained from all participants. We communicated that the interviewee could terminate the interview at any given time or not answer specific questions. Once the interviewee had a clear understanding of the intentions of the study, the researcher asked permission to make an audio recording of the interview solely for translation and note‐taking purposes. To guarantee the interviewee's anonymity, no names or contact information was written down or recorded, and no information was stored that could lead to identifying participants. All files and information collected during the interview were treated as confidential. All research was conducted in accordance with regulations of the national Instituto da Biodiversidade e das Áreas Protegidas (IBAP) and the national Instituto Nacional de Investigação das Pescas e Oceanografia of Guinea‐Bissau (INIPO) (permit #06/10/IBAP/2021). All data were collected and stored securely, conforming to the regulations and guidelines of the University of Groningen.

### Landing‐site surveys

From February to November 2021, a landing‐site survey was initiated in collaboration with INIPO. An enumerator with experience in fisheries research was trained to document shark and ray landings at the Alto Bandim fish market at peak landing times (06:00–09:00, 3 times a week). The enumerator documented the numbers of landed sharks, rays, and teleosts and collected data on the fishing area (i.e., location name, distance from shore, depth), gear specifications (i.e., gear type, length, mesh or hook size, material), and details on shark and ray catches (i.e., species, number of individuals, lengths, sex). This information was used to determine gear use and fishing trip length, describe fishing effort, and determine the length distribution of captured sharks and rays.

### Small‐scale fishing vessels abundance

To determine the number of small‐scale vessels operating within the boundaries of the archipelago and how this has changed over the past decades, we used satellite imagery of the Alto Bandim small‐scale fishing port. We used the historical satellite imagery option in Google Earth Pro 7.3. The resolution of this imagery from January 2007 to December 2023 was appropriate (∼0.5 m/pixel) (imagery sources: Airbus and Maxar Technology) for counting individual small‐scale fishing vessels (∼8–20 m in length) (Appendix S1). We filtered out images in which cloud cover or low resolution hampered vessel visibility. We exported each satellite image (*n* = 95) and used ImageJ 1.53k to crop each image to a standardized bounding box around the port (0.60 × 0.45 km). We then annotated each fishing vessel in this bounding box as a proxy for the number of fishing vessels actively fishing in the Bijagós. Images were available for multiple months for most years, and years for which data from only a single month were available (2008 and 2023) were removed from the analysis (Appendix S5). This approach most likely provided only an estimation of small‐scale fishing vessels from Guinea‐Bissau and did not include vessels from neighboring countries (e.g., Senegal and Guinea). Foreign vessels operate in these waters, but catches are landed in their respective countries. Therefore, these vessels would not have appeared in the satellite imagery. We used 2 methods to estimate the likely activity level of the fleet (i.e., how many of the vessels in the port were used for fishing on a particular day): number of days fishers indicated they fished per week in 2020 and preliminary data from a landing‐site survey initiated in June 2024. In this landing‐site survey, we asked fisheries observers to count the total number of vessels berthed in the Alto Bandim port and to count the number of boats offloading catches.

### Data analyses

Data analyses were conducted in R 4.3.0. We analyzed temporal changes using interview data and mixing models to account for variation in fishers’ responses. We used generalized linear mixed models with a Poisson distribution to examine changes in the number of gear sets of each gear type that fishers had on their boat. We used a negative binomial distribution when overdispersion was determined in the Poisson models. We used a gamma distribution for analyzing changes in gear length and soak times for the gear types fishers used. Beach seine nets were included as small multifilament nets based on their material and mesh size.

We used the same approach to analyze changes in the number of fishing vessels observed by fishers at their fishing sites and the duration of their fishing trips (i.e., how many days did they fish at a location). For all these models, we used year as a numerical fixed effect. To account for the introduced variability by the nonindependence of the 2 response values per fisher (i.e., response for the year when participant started fishing and response for the year when participant stopped fishing or the last year of the study), we included the unique (anonymous) identifier for each fisher (fisher ID) as a random effect.

The number of vessels in the primary small‐scale fishing port was analyzed by modeling the 90% quantile of the vessel count on satellite images. Quantile regression was used to ensure that the maximum number of vessels in the port was modeled for each year, while minimizing the impact of outliers. In this model, we used year as a numerical fixed effect. We then combined these satellite‐based vessel estimates with estimates of how many vessels were active based on the interviews and landing‐site survey.

To determine changes in the abundance of species groups based on fisher experience, we used generalized additive mixed models with a negative binomial distribution to account for overdispersion. In these models, we used the number of individuals of a species group captured per fishing trip as a response variable, year as a (numerical) fixed variable, and fisher ID as a random effect. If fishers provided a range (e.g., 2–4 individuals captured) during the interviews, we used the midpoint for further data analyses. We used the prediction of fishing trip duration as an offset to transform the number of individuals captured per fishing trip to the number of individuals captured per day per vessel. Species group composition was determined for each decade from 1960 to 2020 as the proportion of individuals of a species group captured relative to the total number of sharks and rays captured (as recalled by interviewed fishers). These proportions for each decadal interval were compared using a permutational analysis of variance (i.e., permanova).

The bias of historical estimates by fishers of nontarget species can differ across recall timescales (i.e., catches are estimated higher over short timescales; Aylesworth & Kuo, 2018). To account for extreme values due to overestimations likely caused by recall bias but to retain a large enough data set, we removed the top 5% of the data to minimize the influence of these outliers. Because data points of a fisher were linked (i.e., one data point when the fisher started fishing and when the fisher stopped fishing or in 2020), both data points were removed when one or both were in the top 5% of the data.

We used generalized linear mixed models with a gamma distribution to analyze changes in the mean length of species groups, with year as a fixed variable and fisher ID as a random effect. We removed values below the smallest size at birth and above the maximum reported size for species in each species group to correct for under‐ and overestimation. Statistical model selection was conducted by comparing models based on the Akaike information criterion (AIC) and Bayesian information criterion (BIC).

We extrapolated the number of individuals captured per day by one vessel to the number of individuals captured daily throughout the archipelago by the entire active small‐scale fishing fleet. For this, we used the satellite‐based vessel counts and estimates of fishing fleet activity and combined these with our models estimating species group catches. To account for the uncertainty in the species group models and predictions of vessel numbers in the Alto Bandim fish market, we simulated these models for 1000 Monte Carlo iterations. To determine the influence of fleet activity (i.e., the percentage of vessels counted on satellite imagery that are actively fishing that day), we used 2 bootstrapped estimates (10,000 iterations) for fleet activity: one based on our interviews (i.e., the proportion of weekdays that fishers indicated they were fishing with their vessels) and the other based on preliminary data of a 2024 landing‐site survey (observers counted the active and total number of vessels from June to September 2024). We then multiplied predicted catch per unit effort (CPUE) (individuals per day) for each group of species by the predicted number of active vessels (i.e., total satellite‐based vessel count corrected for both activity levels) for each iteration. This resulted in 2 estimates of daily fleet‐wide catches per species group for 2020 based on the 2 different estimates of fleet activity.

## RESULTS

**FIGURE 2:**
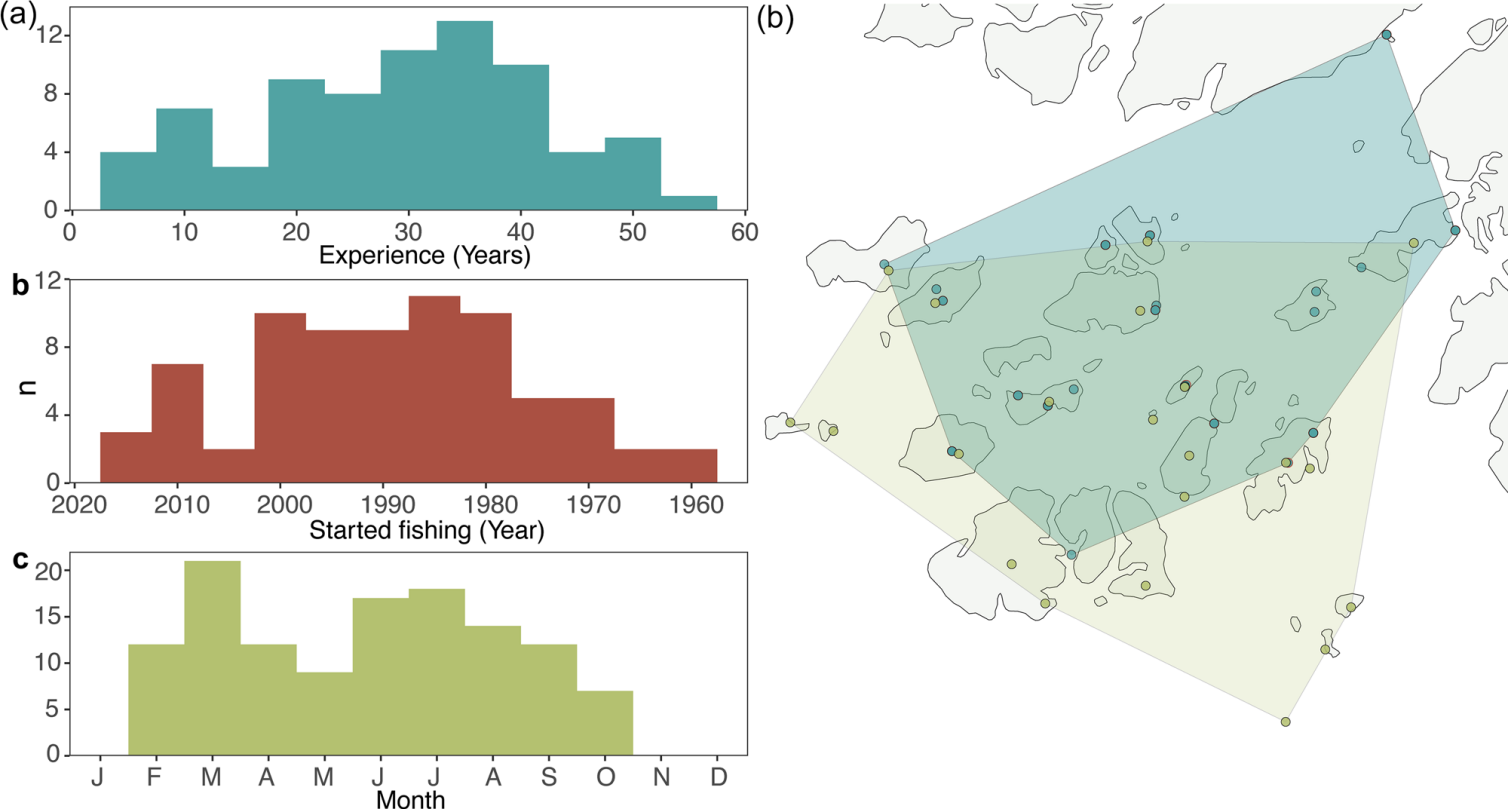
In the Bijagós Archipelago, (a) fishers’ fishing experience in years, (b) year a fisher started fishing, (c) number of vessels sampled each month in landing‐site surveys, and (d) spatial coverage of interviews of fishers (i.e., place of residence of respondents) (blue) and landing‐site survey (i.e., home base of fishing vessels) (green).

From February to June 2021, 75 interviews were conducted with fishers operating throughout the Bijagós Archipelago (Figure 2; Appendix S6). The majority of interviews (82.6%) were conducted in March (*n* = 49) and April (*n* = 13). The fishing experience of fishers ranged from 6 to 56 years (mean [SD] = 29.3 years [12.4]), which corresponded to a retrospective period from 1964 to 2020 (Figure 2a,b). As part of the landing‐site survey, 122 surveys of catch landings were conducted (113 unique vessels that fished throughout the archipelago) (Figure 2c,d). Vessels operating in the archipelago were monitored from February to November 2021, and the highest number of vessels sampled were in March (*n* = 21, 17.2%), June (*n* = 17, 13.9%), and July (*n* = 18, 14.8%) (Figure 2c). Spatially, the combination of interviews and monitoring of the landing site covered fishers and boats of the main islands of the archipelago (Figure 2d).

### Species group trends and composition

**FIGURE 3:**
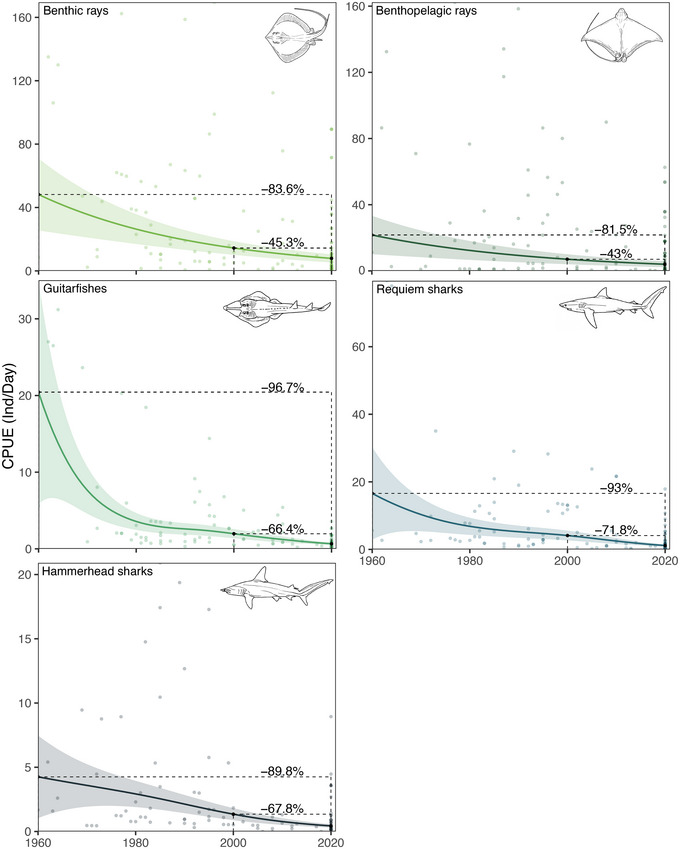
In the Bijagós Archipelago small‐scale fishery, the number of individuals of ray (green) and shark (blue) species groups captured by a single fishing vessel and percent change in individuals caught per day (catch per unit effort [CPUE]) for each species group from 1960 to 2020 and 2000 to 2020 (solid lines, predictions; shading, 95% confidence intervals; black dashed lines, differences between predictions from 1960 and 2000 to the last year of the study, 2020).

Based on the interviews with fishers, we determined that the CPUE significantly decreased for all ray and shark species groups (Figure 3; Appendix S7). Decreases over the entire study period (1960–2020) ranged from 81.5% (95% CI 77.8–82.6) to 96.7% (95% CI 91.4–97.6), whereas decreases from 2000 to 2020 ranged from 43.0 (95% CI 42.4–44.4) to 71.8% (95% CI 69.6–72.8). Although significant declines were noted in CPUE over the study years (*p* < 0.01) (Appendix S7), the most frequently captured elasmobranch group throughout the study period remained the benthic rays, with an estimated mean of 7.88 individuals/day (SE 1.31) captured per vessel in 2020 (*χ* ^2^ = 50.3, *p* < 0.01; Appendix S7).

Overall, the steepest declines (96.7%, 95% CI 91.4–97.6, *χ* ^2^ = 200.3, *p* < 0.001) from 1960 to 2020 were noted for guitarfish, with on average 20.44 (SE 7.45) individuals captured per vessel per day in 1960 and 0.66 (0.08) individuals captured per vessel per day in 2020. Other groups experiencing similar rates of declines over the same period were the requiem (93.0%, 95% CI 72.0–95.0, *χ* ^2^ = 147.0, *p* < 0.001) and hammerhead sharks (89.8%, 95% CI 71.8–92.3, *χ* ^2^ = 123.9, *p* < 0.001). In terms of individuals captured per day, in 2000, fishers caught an estimated 4.12 (0.74) and 1.35 (0.24) individuals of requiem and hammerhead sharks per day, whereas in 2020, this was 1.16 (0.18) and 0.43 (0.07), respectively. This represented a decline of 71.8% (95% CI 69.6–72.8) and 67.8% (95% CI 66.8–68.3) over the last 2 decades for requiem and hammerhead sharks, respectively.

The average size of captured individuals of benthopelagic rays, guitarfishes, requiem sharks, and hammerhead sharks also decreased significantly (Appendix S8). The average guitarfish captured in 1962 was 134.1 cm in total length (TL) (SE 10.1) and 86.7 cm TL (3.9) in 2020 (β = −0.01 ± 0.03, *z* = −4.9, *p* < 0.001). For requiem sharks, it was 148.8 cm TL (14.2) in 1960 and 72.1 cm TL (4.4) in 2020 (β = −0.22 [0.03], *z* = −6.7, *p* < 0.001), and for hammerhead sharks, it was 179.0 cm TL (18.5) and 90.6 cm TL (6.2) (β = 0.21 [0.04], *z* = −5.9, *p* < 0.001).

Generally, size estimates provided by fishers overlapped with known size ranges for the species in these species groups, and fisher's estimates for sizes in 2020 overlapped with size ranges observed during the 2021 landing‐site survey (Appendix S8). Species composition of catches did not differ significantly across decades (df = 5, *F* = 1.0, *p* = 0.3), with rays making up 85.4% (1.7) of the catches over the study period and sharks 14.6% (1.7) (Appendix S9). Based on the landing‐site survey of boats that captured sharks and rays, the highest proportion of elasmobranch catches was the blackchin guitarfish (*G. cemiculus*) (22.6%), milk shark (*R. acutus*) (27.3%), and scalloped hammerhead shark (*Sphyrna lewini*) (7.7%).

### Gear use and fishing effort

**FIGURE 4:**
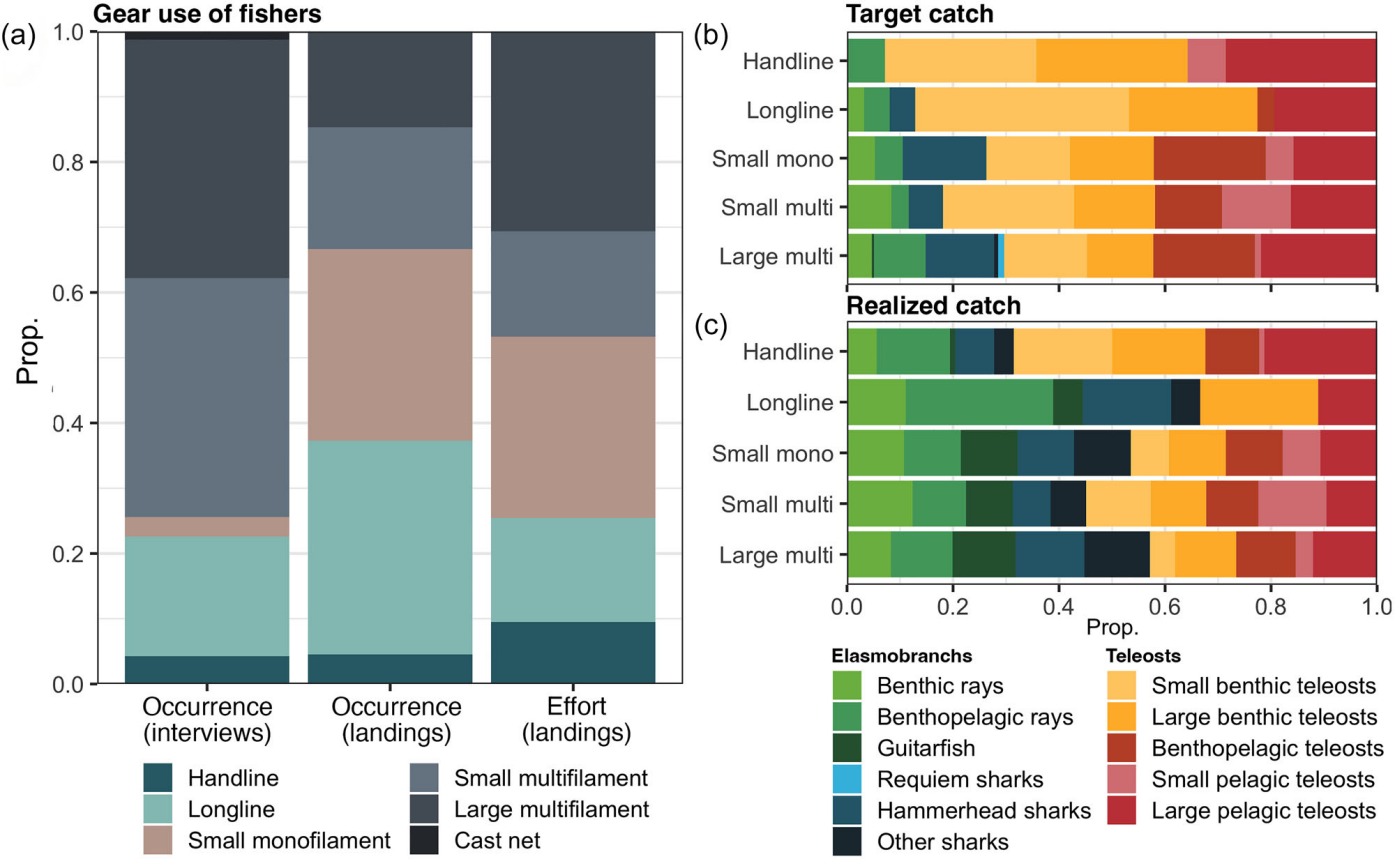
In the Bijagós Archipelago small‐scale fishery, the prevalence (a) of different gear types as a proportion of interviewed fishers who use a particular gear, the occurrence of gear on vessels sampled during the landing‐site survey, and the hours of soak time (effort) a gear was used during fishing trips; (b) of gears used by fishers to target particular species groups (target catch); and (c) of species group captured (realized catch) (mesh sizes >40 mm mesh for large multifilament nets and ≤40 mm for small multifilament and monofilament nets).

Large multifilament (>40‐mm mesh), small multifilament (≤40‐mm mesh), and longlines were the most common gears based on interviews, whereas small monofilament nets (≤40‐mm mesh) were the second‐most common gear based on landing‐site surveys (Figure 4a). Based on these surveys, large multifilament, small monofilament, and longlines were the most prevalent gear types. Target catches predominantly consisted of teleost species groups. However, 29.7% and 26.3% of fishers used large multifilament and small monofilament nets, respectively, to target elasmobranchs (Figure 4b). Fishers mostly used demersal small monofilament nets to target benthic rays. The realized catch (i.e., fishers stating catches of certain species groups with a gear type) showed that elasmobranchs were captured using all gear types, but mostly with longlines (66.7%), small monofilament (53.6%), large multifilament (57.1%), and small multifilament nets (45.1%) (Figure 4c). The mean soak time of large multifilament nets significantly increased by 25.3%, from 5.9 (95% CI 3.1–8.7) hours in 1960 to 7.9 (95% CI 4.8–11.3) hours per deployment in 2020 (β = 0.09 [SE 0.03], *z* = 3.22, *p* = 0.001). However, no significant changes in the number of sets, gear length, and soak times were reported for most gear types (Appendix S10).

**FIGURE 5:**
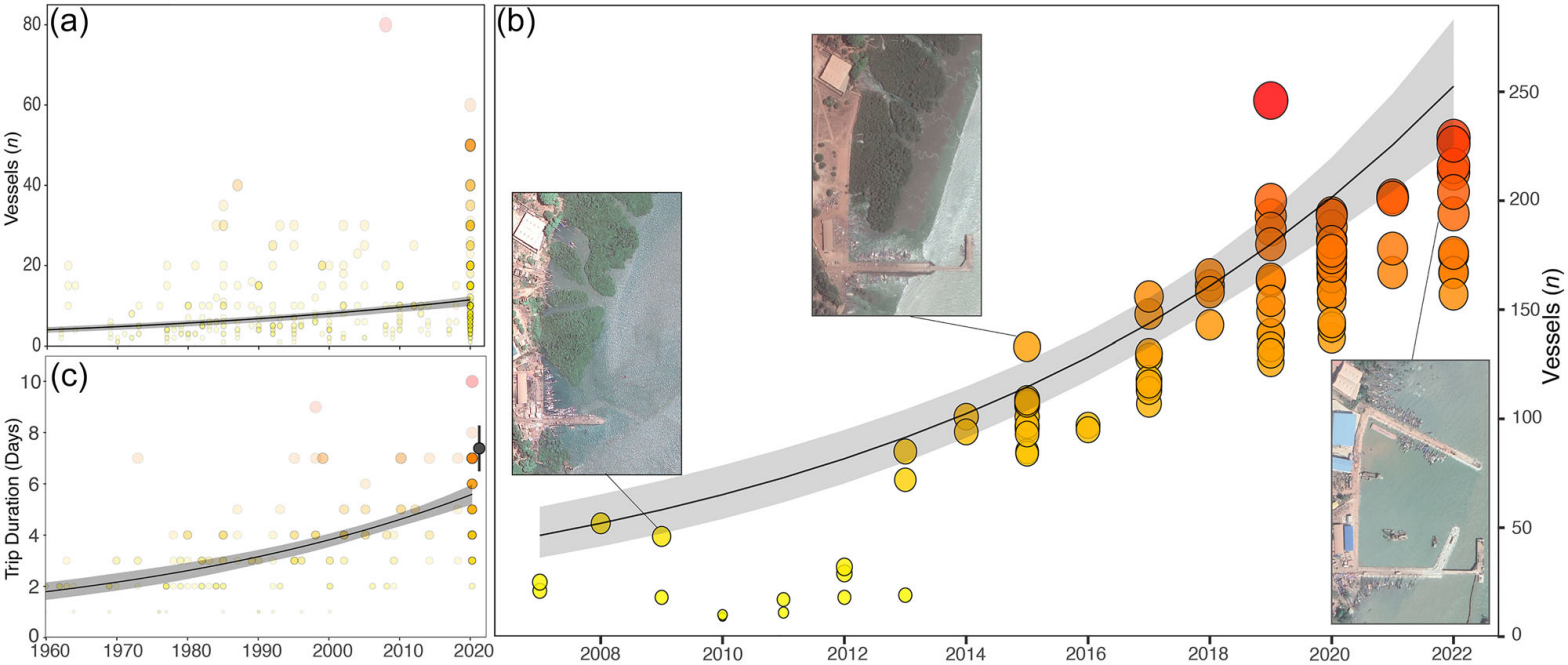
In the Bijagós Archipelago small‐scale fishery, over time (a) fisher estimates of how many vessels they observed in their fishing area, (b) change in the number of small‐scale fishing vessels and expansion of the primary port of Alto Bandim in Bissau (satellite images) (curve, 90% quantile regression; shading, 95% confidence interval), and (c) fisher reports of fishing trip duration in the 2021 landing‐site survey (black point, 95% confidence interval). Satellite images are from Google Earth Pro (downloaded on 2 September 2023).

Fishers indicated that the number of vessels that they observed at their fishing locations increased from 4.0 vessels (SE 0.4) in 1960 to 11.5 (0.6) in 2020 (β = 0.31 [0.03], *z* = 11.07, *p* < 0.001) (Figure 5a), representing an increase of 187.5%. The total number of small‐scale fishing vessels operating in the archipelago increased by a mean of 12.0% (1.1) on an annual basis and by a total of 443.7% from 2007 (46.4 [5.9]) to 2022 (252.5 [14.8], β = 0.11 [0.01], *t* = 9.93, *p* < 0.001) (Figure 5b). Furthermore, fishers indicated that the duration of their fishing trips increased from 1.8 (1.5–2.1) days in 1960 to 5.6 (5.2–6.0) days in 2020 (β = 0.35 [0.03], *z* = 10.38, *p* < 0.001) (Figure 5c). Based on the landing‐site survey, fishing vessels catching sharks and rays were out at sea for a mean of 7.4 days (0.5) in 2021 per fishing trip (Figure 5c). Fishers indicated that they were active throughout the year, and we found no differences in seasonal fishing effort based on the interviews (Appendix S11).

### Predicting daily fleet‐wide catches

**FIGURE 6:**
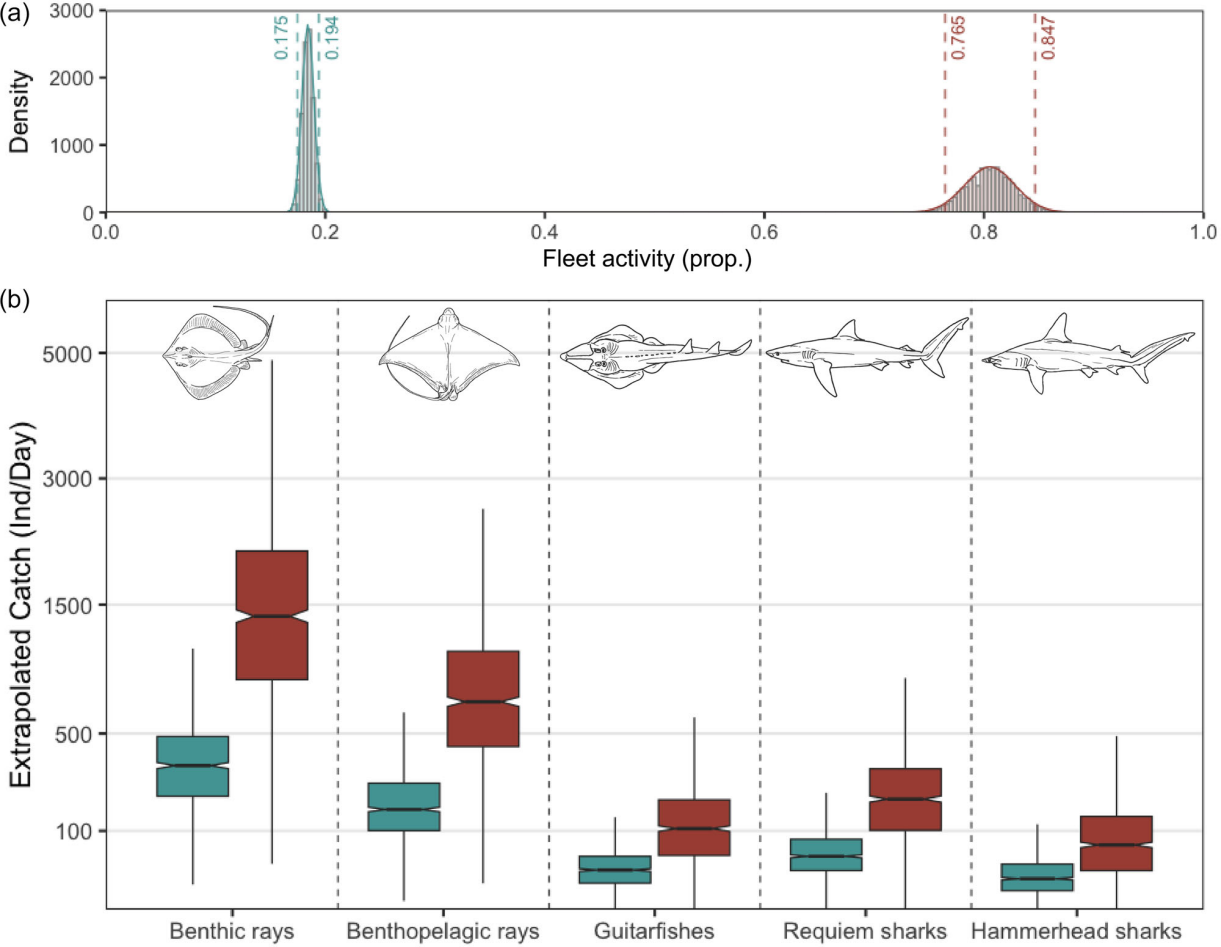
(a) Fleet activity (proportion of fleet deployed for fishing) based on fisher reports of days per week they fished (red) and counts of active and total vessel numbers from June to September 2024 (blue) (bootstrapped estimates) and (b) extrapolated daily catches of the small‐scale fishing vessel fleet for each species group (blue, low estimates based on the lower estimates of fleet activity derived from the preliminary fishing observer program; red, high estimates based on the higher estimates of fleet activity as reported by fishers). Catch is transformed to the square root.

Estimates of the current number of individuals of each species group captured on a single day in the last study year, 2020, ranged from 61 to 264 sharks and 522 to 2194 rays depending on the estimates of fleet activity (Figure 6). Based on our interviews, we estimated that the mean fishing vessel activity was 80.5% (95% CI 76.5–84.7) of the total number of vessels (Figure 6a). However, based on preliminary counts of active vessels, this estimate was around 18.4% (95% CI 17.5–19.4) (Figure 6a). Under this lower estimate for fleet activity, we extrapolated daily fleet‐wide catches to 335 (median) individuals/day (interquartile range [IQR] 207–484) of benthic rays, 25 individuals/day (IQR 11–46) of guitarfishes, and 15 individuals/day (IQR 6–34) of hammerhead sharks (Figure 6b). Under the higher (i.e., interview‐based) estimate of fleet activity, these numbers (and their variation) increased to a median of 1391 individuals/day (IQR 856–2104) of benthic rays, 106 individuals/day (IQR 47–195) of guitarfishes, and 67 individuals/day (IQR 25–140) of hammerhead sharks (Figure 6b).

## DISCUSSION

We showed that a novel combination of readily available approaches can be used to shed light on trends in SSF and their historical catches of vulnerable species, such as sharks and rays. Our findings indicated severe declines in landings of all shark and ray species groups (83–97% for 1960–2020) in the Bijagós Archipelago, Guinea‐Bissau. Simultaneously, the size of the fishing fleet continued to increase exponentially. Although recent catches and landings were substantial (daily fleet‐wide catches are approximately 61–264 sharks and 522–2194 rays), they were only a fraction of reported historical catches despite no noteworthy changes in gear use. A decrease in CPUE and a reduction in average sizes for shark and ray species groups, as we reported here, suggest a corresponding decrease in the populations of these species groups. This is concerning considering the threatened status of most shark and ray species found in Guinea‐Bissau and the limited fisheries management and enforcement in place.

Globally, sharks and rays face increasing threats with overexploitation as the primary driver behind their deteriorating status (Dulvy et al., 2021). The conservation status of sharks and rays in the West African region has been challenging to assess due to data deficiency. However, available species‐level information indicates severe declines. For example, based on 3 fisheries data sets from Mauritania, the reduction of the common guitarfish (*R. rhinobatos*) was estimated to be 85% over 42 years (Jabado, Pacoureau, et al., 2021). Similarly, the reduction of the common smooth‐hound shark (*Mustelus mustelus*) was estimated to be 92.4% over 53 years (Jabado, Chartrain, et al., 2021). Overall elasmobranch CPUEs in the region have declined by 44% from 1981 to 2015 (Pauly et al., 2020).

Our findings suggest that this is not limited to a few species but that all species groups have deteriorated. The negative population trends of guitarfishes and hammerhead sharks are especially worrying because these are among the most threatened vertebrates globally (Dulvy et al., 2021; Kyne et al., 2020). In other West African areas, guitarfish and hammerhead sharks are among the most captured species despite their deteriorating international conservation status (Moore 2017, Dia et al., 2023; Doherty et al., 2023).

Although our LEK approach resulted in large uncertainties around estimated catches, our results showed a decline in shark and ray catches. These results are consistent with findings in other coastal areas where SSF are predominant and have also reported declines in historical elasmobranch catches (i.e., 37.6–87.7% declines) or size (i.e., size reductions of 29.9–49.5%) (e.g., Dia et al., 2023; Fernando & Stewart, 2021; Humber et al., 2017; Kyalo & Stephen, 2013; Lemrabott et al., 2024b; Wambiji et al., 2022). In the absence of changes in fishing practices (e.g., fishing gears, fished areas), declines in CPUE and average size are assumed to be indicators of overfishing (Froese, 2004; Hoggarth et al., 2006) and were reported 2 decades ago in this region (Diop & Dossa, 2011). Our estimates of elasmobranch catches may be an underestimation because fishers report that many vessels from neighboring countries (e.g., from Senegal and Guinea) target sharks and rays in the archipelago and may land their catches in these countries (Campredon & Cuq, 2001; Diop & Dossa, 2011). Further, we did not account for industrial vessels operating in the waters of Guinea‐Bissau that may have large elasmobranch catches (Leurs et al., 2024a). Overall, this suggests that current fishing pressure on sharks and rays is likely higher.

Declines in shark and ray populations can affect the ecological functioning of coastal areas (Dedman et al., 2024). Sharks and rays can have a large variety of food web roles (Hammerschlag et al., 2019; Heithaus et al., 2022; Navia et al., 2016). We found that the average size of most elasmobranch species groups declined, which could be indicative of intra‐ and interspecific changes in the elasmobranch community. A decrease in the average total length of a species group (e.g., Carcharhinidae) could be caused by the removal of large (adult) individuals or by a shift in species composition toward smaller species. Large, slow‐growing elasmobranch species are typically affected more by fisheries compared with smaller, more productive species (e.g., Dulvy et al., 2021; Walls & Dulvy, 2020). A shift from large to smaller elasmobranch species has been documented elsewhere (e.g., Haque et al., 2021a; Jaiteh et al., 2017) and may be caused by higher intrinsic growth of smaller species or release from predation or competition in the absence of large species or conspecifics (Dedman et al., 2024; Dulvy et al., 2000). Our estimates of sizes based on LEK showed high variation but overlapped with the size ranges observed in the landing surveys.

Changes in the community composition, or even a complete loss of species groups (e.g., guitarfish), could lead to a loss of ecological roles and ecosystem impairment. Fisher's LEK indicated that species once common, such as sawfishes, have disappeared from West Africa (e.g., Leeney & Poncelet, 2015). Research in neighboring Mauritania and Senegal suggests that wedgefishes and some species of guitarfishes have been extirpated (R.W.J., unpublished data; Dia et al., 2023). Community elders in the Bijagós also indicated that they are worried that *kasapai* (guitarfishes) face the same fate (G.L., unpublished data). It was possible to collect detailed information on some species or distinct species groups due to distinct morphological features that fishers referred to (i.e., rostrum of sawfishes, coloration, and large fins of wedgefishes) (Jabado et al., 2015). Although declines at the group level were possible to estimate, the lack of species‐specific information in our study may have masked more significant within‐group declines of species that fishers could not accurately distinguish (e.g., differentiating milk sharks from other carcharhinids). Further research is needed to determine changes in the regional elasmobranch composition.

The disappearance of sharks and rays from these coastal areas could also have socioeconomic repercussions for coastal communities. Our results suggest that fishers go to sea more often or for extended periods, yet catch less. This aspect of overfishing can have significant implications for local incomes and subsistence (Golden et al., 2016). Shark fisheries are often linked to local consumption of shark and ray meat, and shark fisheries can form a crucial part of local economies (Booth et al., 2019; Glaus et al., 2018; Karnad et al., 2020; Sall et al., 2021; Seidu et al., 2022), especially in regions with high poverty levels and low food security (Golden et al., 2016). Although declines in target species themselves are problematic for fishers, regulating and managing (shark) fisheries may also impede local communities’ ability to address their basic needs (Booth et al., 2019). Regulating shark fisheries will therefore need to go hand in hand with efforts to alleviate poverty and secure communities’ basic needs, as well as safeguarding local sociocultural values. In the Bijagós Archipelago, sharks and rays have a central role in spiritual ceremonies and traditions (Cross, 2014; Diop & Dossa, 2011; Leeney & Poncelet, 2015). The sawfish features on the regional currency (West African CFA Franc), villages have buildings and ornaments inspired by this species, and sawfish, guitarfish, and hammerhead sharks are often represented in traditional masks and costumes during ceremonies (Cross, 2014; Leeney & Poncelet, 2015). The loss of the sawfish also represents a loss of the cultural aspects of these species. These socioeconomic and ecological considerations make the management of small‐scale (shark) fisheries complex yet important (Booth et al., 2019; Haque et al., 2021b; Sall et al., 2021; Seidu et al., 2022).

We found that current fleet‐wide catches of sharks and rays remain high, although our estimates are based on large differences in estimated fleet activity from the 2 methods used, which resulted in a large variation in extrapolated catch estimates. A reduction in the small‐scale fishing fleet through regulation and enforcement of fishing permits would likely reduce catches of sharks and rays. The limitation and monitoring of fleet size have been used in other coastal areas to reduce fishing effort on species of concern, or to regulate overall fishing effort (Lemrabott et al., 2024a; Salas et al., 2007). Still, these measures can only be justified and sustainable for long‐term impact if alternative incomes are realized and the livelihoods of fishing communities are safeguarded (Pomeroy, 2012; Salas et al., 2007). Although MPAs can also be an effective strategy to conserve shark and ray species, for larger and mobile elasmobranch species, protected areas may not be as beneficial (Mackeracher et al., 2019; White et al., 2017). Our results showed that fishing pressure and associated catches of sharks and rays throughout the archipelago remain high, including in the MPAs of Orango and the community‐managed MPA of Urok. Improving enforcement of existing regulations and limiting fishing effort by reducing fleet sizes will likely benefit these vulnerable populations. This is particularly important because these large coastal areas are mainly used by early‐life‐stage elasmobranchs with relatively small home ranges (Knip et al., 2010; Leurs, Nieuwenhuis, et al., 2023a).

The Bijagós Archipelago is likely to be an important area for early‐life‐stage elasmobranchs (e.g., *G. cemiculus* and *S. lewini* [G.L., unpublished data), as are similar coastal areas in the region (e.g., the Banc d'Arguin in Mauritania; Dia et al., 2023). However, other strategies to minimize continued exploitation of these vulnerable species should also be developed, implemented, and further studied. This includes enforcing and extending the monofilament net ban in‐ and outside the protected areas, a retention ban of critically endangered species like hammerhead sharks and guitarfish, gear‐length restrictions, and seasonal closures of areas where fishing effort and key areas for elasmobranchs overlap (e.g., reproductive areas, important shark and ray areas [Hyde et al., 2022]). The latter should be studied further because the presence of some elasmobranch species is likely linked to the rainy season (Leurs, Verkuil, et al., 2023). In conjunction with improved actions to support conservation of these species and to measure their impact and effectiveness, a monitoring system of catches of sharks and rays in SSF is essential.

Our reconstructed catches relied on fisher LEK, satellite‐based vessel counts, and landing surveys. LEK has become a key component of biology (e.g., Anadón et al., 2009; Gilchrist et al., 2005; Neis et al., 1999) and to the study of distribution (Lopes et al., 2019) and temporal changes of species (Beaudreau & Levin, 2014; Gilchrist et al., 2005). The use of LEK also ensures the inclusion of resource users in research and decision‐making and can lead to a broader understanding of the socioecological system (Beaudreau & Levin, 2014; Gilchrist et al., 2005; Lopes et al., 2019). However, effective management also requires quantitative information (Gilchrist et al., 2005; Tesfamichael et al., 2014). LEK studies can be limited to the collection of qualitative (Gilchrist et al., 2005) or low‐resolution quantitative information (Anadón et al., 2009; Neis et al., 1999; Silvano & Valbo‐Jørgensen 2008). In many cases, quantitative information is also collected at vague temporal scales that are difficult to recall (e.g., abundance for each decade; Azzurro et al., 2011; Beaudreau & Levin, 2014; Colloca et al., 2020). The outcomes can be highly variable or lack appropriate resolution, limiting adequate inclusion of LEK results in management (Hind 2015).

To improve the quality of the estimates obtained from our LEK surveys, we focused on the moments a fisher can likely recall best: when the fisher started fishing and the fisher's current situation (Tesfamichael et al., 2014). This method can be used to estimate temporal change when combined with a sampling scheme that targets fishers across the entire age range of the fishing community and spatial scale of the study area and when the sample size of fishers is representative of the fishing community size.

Using this method, we found severe declines of all elasmobranch species groups and that younger fishers are likely used to catching fewer elasmobranchs than older fishers. This ecological baseline shift (Pauly, 1995) is similar to the change in generational sawfish baselines (Braulik et al., 2020; Leeney & Poncelet, 2015). However, as our uncertainty levels around catch estimates show, fisher LEK studies can be affected by errors and variability introduced by willingness to share information (e.g., linked to noncompliance to regulations; Anadón et al., 2009), differing stakeholder experiences and interpretation, and the ability to identify species of concern (Anadón et al., 2009). We observed inconsistencies in the use of monofilament (which is prohibited) between the interviews (i.e., limited use) and the landing‐site survey (i.e., more commonly used). We established a mutual understanding of elasmobranch species through the use of species photographic cards. Fishers often have accurate knowledge of species identification, especially species that are easily recognizable or closely linked to communities, as with sharks and rays in the Bijagós (Jabado et al., 2015; Neis et al., 1999).

Our satellite‐based vessel counts may have been affected by on‐site anchorage of vessels or the limited availability of satellite imagery. Furthermore, the development of the port may have resulted in vessel count bias. Long‐term landing‐site monitoring is necessary to confirm our estimates of fleet activity and catches and to investigate possible seasonal influences in this fishery that could not be confirmed using our LEK approach. Our estimates of fleet activity based on 2 different methods differed significantly, and future research should determine the true fleet activity levels. These differences in estimated fleet activity can be caused by methodological differences, where one method (interviews) relied on fishers being able to recall fishing activities and the other relied on a pilot port‐based vessel count at the start of the rain season. Therefore, our low (18%) estimate of fleet activity may be influenced by seasonal changes in fishing effort, and our high (80%) estimate may be influenced by observer or recollection bias. Results of previous studies of SSF in Guinea‐Bissau and West Africa show seasonality in catches and fishing effort (Campredon & Cuq, 2001; Cross 2014; Sall et al., 2021).

We found that a combination of LEK and conventional methods (e.g., landing surveys and satellite boat counts) can provide important baseline information on sharks and rays for areas where this information is lacking, but these methods can generate large uncertainties and results need to be validated based on conventional methods (e.g., long‐term landing surveys, registration systems of vessels). Our reconstructed declines in catches and average size ranges of shark and ray species groups—with no measurable changes in gear use and increasing effort—are consistent with patterns reported elsewhere in the region and suggest alarming population declines due to overexploitation. This information is needed to improve the management of threatened marine species, especially in regions with limited resources and capacity, and should serve as the basis for future (adaptive) management and further research of these vulnerable species. These species have both ecological and socioeconomic importance to coastal communities, such as in the Bijagós Archipelago, and our results give a first indication of their status. Considering the current status of sharks and rays in the region, immediate action needs to be taken to reduce fishing mortality through improved fisheries monitoring and management (Dulvy et al. 2017), enforcement of existing regulations (e.g., monofilament bans) targeting elasmobranch catches, regulation of fishing fleet sizes, live release of elasmobranchs, and delineation of areas important for shark and rays in combination with seasonal closure of fisheries. However, these steps can be taken only by addressing the complex interplay of these fisheries and the needs of local communities and, therefore, should be done concurrently with the development of alternative incomes, improvement of basic needs, and strengthening of local research capacity.

## Supporting information



Supporting Information
