# Supplementary material for: Reconstructing historical catch trends of threatened sharks and rays based on fisher ecological knowledge
Source: Conserv Biol. 2025 May 31;39(5):e70059. doi: 10.1111/cobi.70059 (PMC12451511; doi:10.1111/cobi.70059)

**Appendix S1 - Fishing vessel types**

The two predominantly used vessels in the small-scale fishery within the Bijagós Archipelago are the dug-out canoe (A; approximately 5 to 8 meters in length) and the larger pirogue (B; ~15 meters in length). Whereas the dug-out canoes are mostly solely human-powered, the pirogues are used mainly by one or multiple outboard engines.


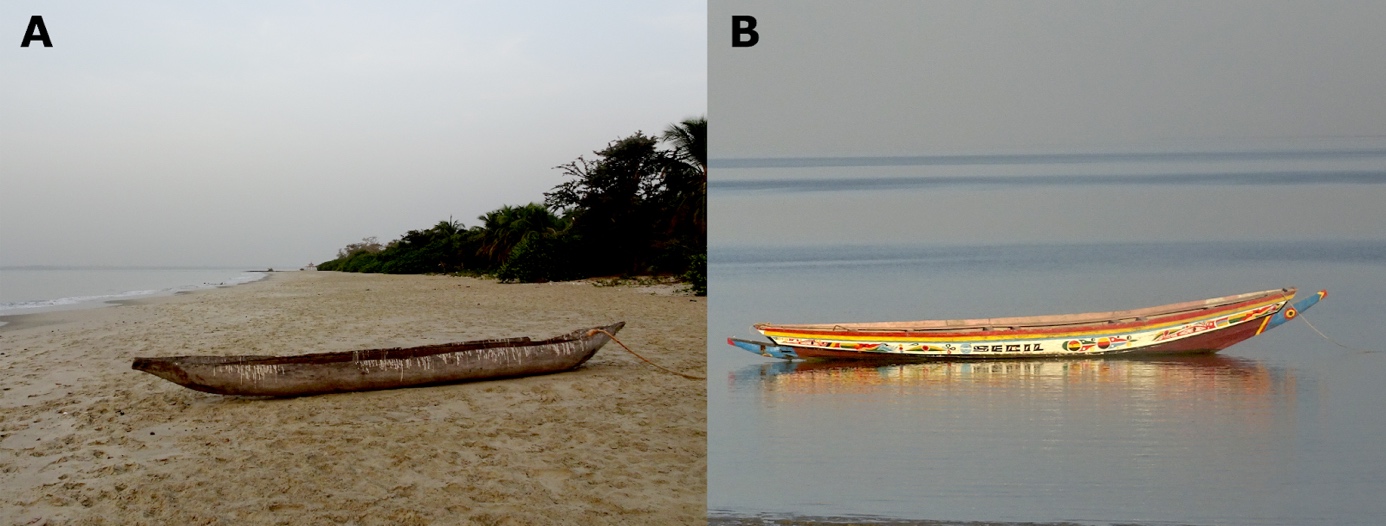


**Appendix S2 - Interview questionnaire (translated from Portuguese)**

| **A. General information** | | | |
| --- | --- | --- | --- |
| Date: |  | Interviewers: |  |
| Start time: |  | Notetaker: |  |
| End time: |  | Location: |  |
| Notes/comments: | | | |
| **B. Demography** | | | |
| B1.a | From which Island are you? |  | |
| B1.b | Have you always fished on this island or have you ever moved? |  | |
| B1.c | Since how long have you been fishing? |  | |
| B1.d | Are you still active/when did you stop? |  | |
| **C. Technical information** | | | |
| **C1. Vessel specifications** | | | |
| C1.a | What type of boat do you use (e.g. pirogue, canoe, metal boat)? |  | |
| C1.b | What is the power of the boat engine (horsepower, sails)? |  | |
| C1.c | How long is the boat (in meters)? |  | |
| C1.d | Approximately how many boats are operational on your island? |  | |

| **C2. Specifications of fishing material**; *Starting with the fishing gear you use most, on an average fishing trip, what types of fishing gear do you use?* | | | | | | | | | | | | |
| --- | --- | --- | --- | --- | --- | --- | --- | --- | --- | --- | --- | --- |
| **#** | | ***Type of fishing gear*** | |  | | | | | | **In the year past** | | **When started fishing** |
| C2.a  (*5x to C2.e*) | |  | | How many sets of this fishing gear do you have on your boat? | | | | | |  | |  |
|  |  |  |  | Can you tell me how many hooks, the mesh size, the net size, the height of the net, etc.? | | | | | |  | |  |
|  |  |  |  | What material is it made of? | | | | | |  | |  |
|  |  |  |  | What do you use this fishing material for (which species)? | | | | | |  | |  |
|  |  |  |  | On an average fishing trip, how long do you leave your fishing gear in the water? | | | | | |  | |  |
| **C3. Specifications of the fishing area**; *Starting with the area you go to the most, can you show me where you fish on the map? And what are the names of these areas?* | | | | | | | | | | | | |
| **#** | | ***Fishing area*** | | | | **For each fishing area, indicate:** | | | | **In the year past** | | **When started fishing** |
| C3.a  (*7x to C3.g*) | |  | | | | Why are you going to this area? (or why not anymore?) | | | |  | |  |
|  |  |  |  |  |  | What months of the year do you go here? | | | |  | |  |
|  |  |  |  |  |  | On average, how many hours do you fish per week here? | | | |  | |  |
|  |  |  |  |  |  | Approximately how many fishers fish in this area? | | | |  | |  |
| **C4. Species- specific information.** | | | | | | | | | | | | |
| **#** | | ***Teleost species*** | | |  | | | | | **In the year past** | | **When started fishing** |
| C4.a  (*4x to C4.d for each teleost group*) | | Group 1  (photo 63, 67, 64, 78, 65, 61)  Group 2  (photo 69, 82, 50, 72)  Group 3  (photo 53, 52)  Group 4  (photo 55, 56)  Group 5  (photo 79, 75, 77) | | | How many do you catch per trip (individuals)? | | | | |  | |  |
|  |  |  |  |  | How many kilograms of this species group in total per trip? | | | | |  | |  |
|  |  |  |  |  | What is their average length (in cm)? | | | | |  | |  |
|  |  |  |  |  | Where do you catch these species? | | | | |  | |  |
|  |  |  |  |  | Which gear do you use to catch this species? | | | | |  | |  |
|  |  |  |  |  | In which months do you catch this species? | | | | |  | |  |
|  |  |  |  |  | In which months do you not catch this species? | | | | |  | |  |
| **#** | | ***Elasmobranch species*** | | |  | | | | | **In the year past** | | **When started fishing** |
| C4.f  (*5x to C4.j for each group*) | | Group 6  (photo 12, 2)  Group 7  (photo 9, 13, 8, 11)  Group 8  (photo 19)  Group 9  (photo 9)  Group 10  (photo 26, 27, 90) | | | How many do you catch per trip (individuals)? | | | | |  | |  |
|  |  |  |  |  | How many kilograms of this species group in total per trip? | | | | |  | |  |
|  |  |  |  |  | What is their average length (in cm)? | | | | |  | |  |
|  |  |  |  |  | Do you process the fish in any way (e.g., cleaning)? How do you sell them (e.g., whole, without tail, in parts)? | | | | |  | |  |
|  |  |  |  |  | What is or was the price per kilo? And who do you sell it to? | | | | | Whole body:  Meat:  Cartilage:  Fins:  Liver:  Skin: | | Whole body:  Meat:  Cartilage:  Fins:  Liver:  Skin: |
|  |  |  |  |  | Why do you catch this species? | | | | |  | |  |
|  |  |  |  |  | Where do you catch these species? | | | | |  | |  |
|  |  |  |  |  | Which gear do you use to catch this species? | | | | |  | |  |
|  |  |  |  |  | In which months do you catch this species? | | | | |  | |  |
|  |  |  |  |  | In which months do you not catch this species? | | | | |  | |  |
| **C5. Species that disappeared** | | | | | | | | | | | | |
| ***#*** | | | **Did you ever catch or still catch this species?** | | | | **Why do you think this species is no longer caught?** | | **When was the last time you caught this species?** | | **Where was this?** | |
| C5.a Photo 43 | | |  | | | |  | |  | |  | |
| C5.b Photo 31 | | |  | | | |  | |  | |  | |
| C5.c Photo 18 | | |  | | | |  | |  | |  | |
| C5.d Photo 17 | | |  | | | |  | |  | |  | |
| **D. Additional questions** | | | | | | | | | | | | |
| D1. Open questions | | | | | | | | | | | | |
| D1.a | What are the biggest challenges in your daily life as fisher? | | | | | | |  | | | | |
| D1.b | Is there anything else you want to share with us? | | | | | | |  | | | | |
|  |  |  |  |  |  |  |  |  |  |  |  |  |

**Appendix S3 - Comparison between traditional decade-long surveys and surveys focusing on the ‘most memorable moments’.**

Conceptual comparison of traditional interview methods to elucidate fisher ecological knowledge (left), compared to the method we describe addressing only the most memorable moments in the fisher’s career (right), when one started fishing and the current situation (or the year one stopped). This figure consists of conceptual data to clarify the methodology used, described in more detail in Tesfamichael *et al.* (2014).


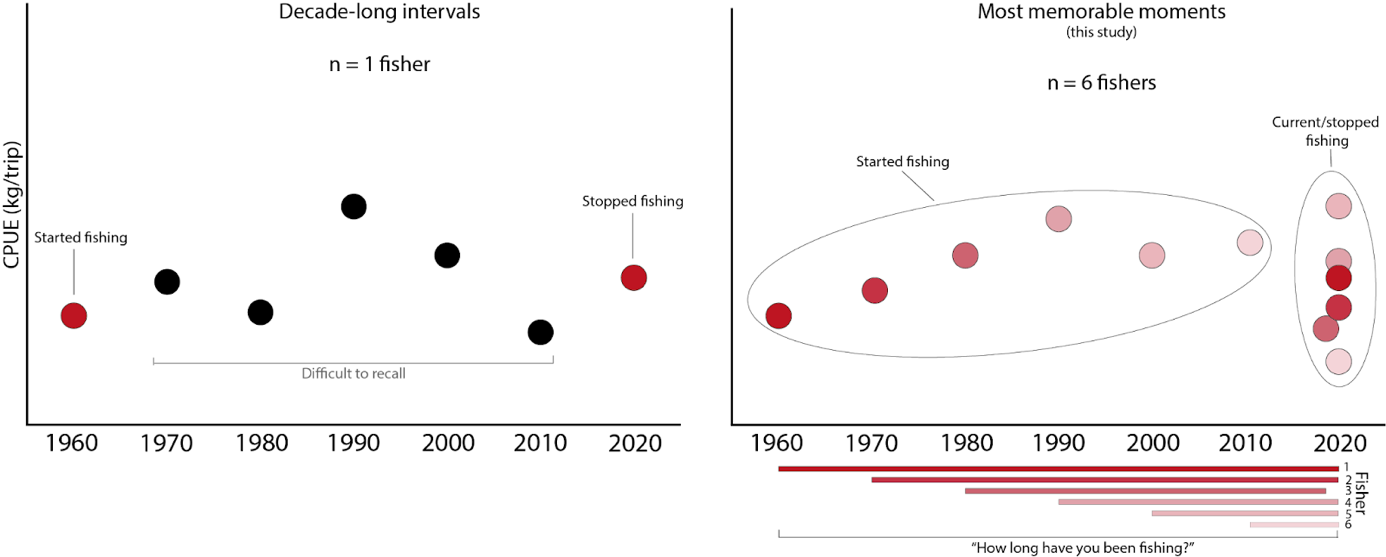


**Appendix S4 - Species group classification**Specification of species groups and the species included in each, with their respective scientific, Creole, Bijagó, and English names.

| **Species group** | **Scientific name** | **Creole name** | **Bijagó name** | **English name** |
| --- | --- | --- | --- | --- |
| Benthic rays | *Hypanus spp.*  *Dasyatis spp.*  *Fontitrygon spp.*  *Gymnura spp.* | Pis reia  Pis reia  Pis reia  Pis reia | Ebala  Ebala  Ebala  Ebala ebenten | Stingrays  Stingrays  Whiprays  Butterfly rays |
| Benthopelagic rays | *Aetomylaeus bovinus*  *Rhinoptera marginata* | Pis manjoty  Pis pumba | Ebala-ecota | Bull ray  Lusitanian cownose ray |
| Guitarfishes | *Rhinobatos spp.*  *Glaucostegus cemiculus* | Kasapai  Kasapai | Esapai  Esapai | Guitarfishes  Blackchin guitarfish |
| Requiem sharks | *Carcharhinus spp.*  *Rhizoprionodon acutus* | Caudo  Caudo | Narangui  Narangui | Requiem sharks  Milk shark |
| Hammerhead sharks | *Sphyrna spp.* | Pis berga |  | Hammerhead sharks |
| Small benthic teleosts | *Eucinostomus melanopterus*  *Pomadasys jubelini*  *Pomadasys rogerii*  *Lethrinus atlanticus*  *Mugil spp.*  *Galeoides decadactylus*  *Pagrus caeruleostictus* | Pis prata  Corcor  Corcor  Simpoti  Tainha  Barbinhu  Sinapa | Nikindima  Ecoli  Ecoli  Umsinpoti  Cacandja  Edohc  Xinapa | Flagfin mojarra  Sompat grunt  Pigsnout grun  Atlantic emperor  Mullet  Lesser African threadfin  Bluespotted seabream |
| Large benthic teleosts | *Arius spp./Calarius spp.*  *Epinephelus aeneus*  *Psettodes belcheri*  *Lobotes surinamensis* | Bagre  Garoupa  Pis bande  Bentana de mar fora |  | Sea catfishes  White grouper  Spottail spiny turbot  Tripletail |
| Benthopelagic teleosts | *Alectis alexandrina*  *Caranx spp.* | Prato de aluminio  Sereia | Caicu  Edene | Alexandria pompano  Jacks |
| Small pelagic teleosts | *Ethmalosa fimbriata*  *Sardinella spp.* | Djafal  Yaiboi | Calapad  Calapad | Bonga shad  Sardinella |
| Large pelagic teleosts | *Pseudolithus elongatus*  *Scomberomorus tritor*  *Sphyraena spp.* | Djoto  Cachureta  Bicuda | Exaló  Caxuleta  Cató | Bobo croaker  West African Spanish mackerel  Barracuda |

**Appendix S5 - Availability of satellite imagery over the study years**

For each year, multiple satellite images were available for multiple months (orange). Images from one month are available only for the years 2008 and 2023. As for 2023, new images were unavailable at the time of data analysis, so we excluded this year from the analysis. The sample size (n) for each year is indicated as the number of available satellite images (i.e., appropriate resolution and no cloud cover).


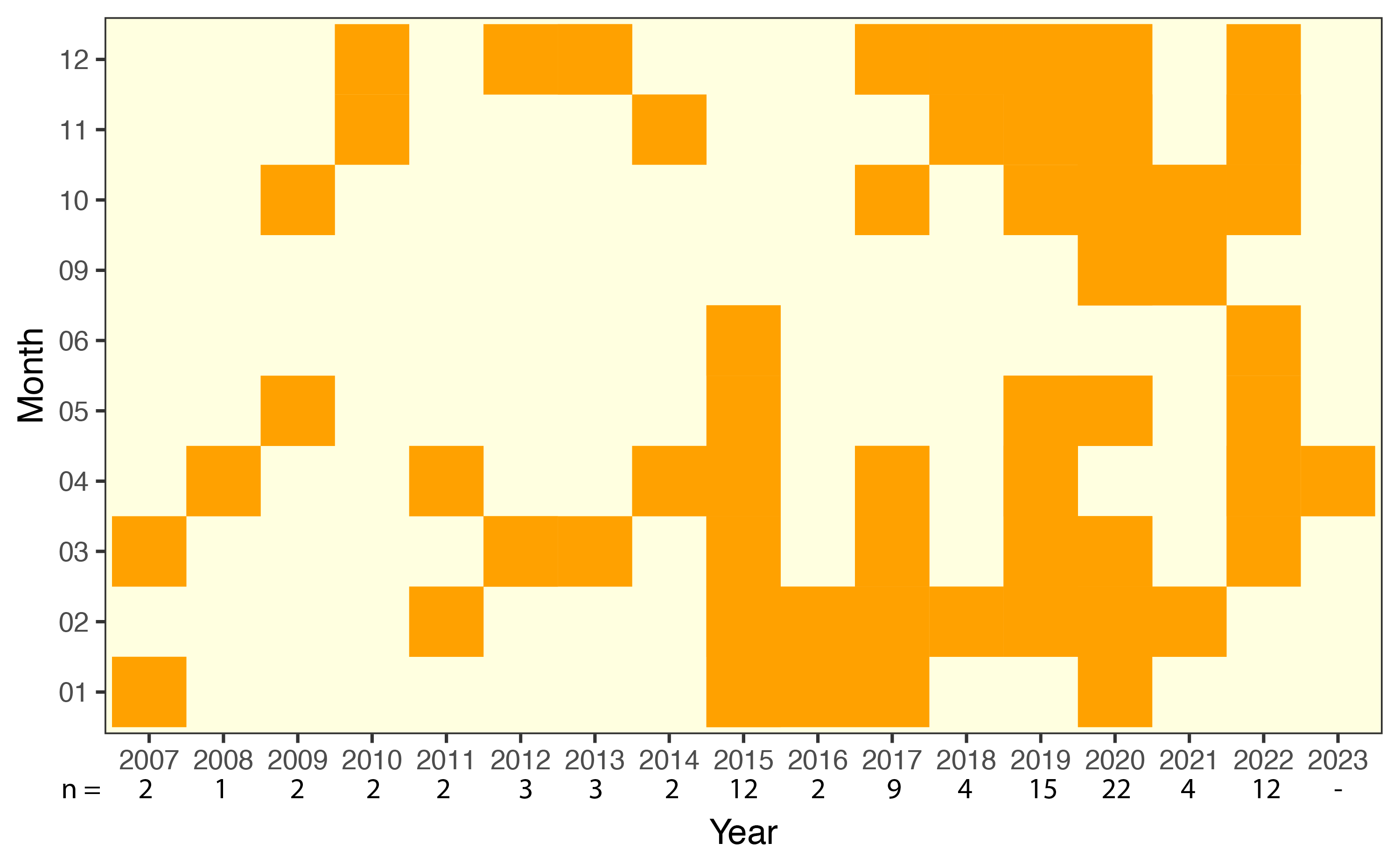


**Appendix S6 - Interview details**Overview of the residence (island) and experience (in years) of interviewed fishers.

| **Region** | **Interviews (N)** | **Fisher experience (years)** |
| --- | --- | --- |
| Bolama | 5 | 10 - 49 (28.8 ± 16.3) |
| Bubaque | 4 | 30 - 47 (38.5 ± 7.5) |
| Canhabaque | 7 | 25 - 56 (36.3 ± 11) |
| Caravela | 5 | 10 - 35 (22.2 ± 11.3) |
| Galinhas | 3 | 6 - 36 (20 ± 15.1) |
| João Vieira | 0 | - |
| Orango | 1 | 31 |
| Soga | 2 | 21 - 38 (29.5 ± 12) |
| Uno | 13 | 6 - 52 (29.9 ± 13) |
| Mainland | 11 | 10 - 40 (23.9 ± 10.5) |
| Abroad | 2 | 7 |
| **Total** | **75** | **6 - 56 (29.3 ± 12.4)** |

**Appendix S7 - Species group model diagnostics.**

| **Species Group** | **Model structure** | **AIC** | **BIC** | **R_2_** | **X^2^** | **p** |
| --- | --- | --- | --- | --- | --- | --- |
| Benthic rays | Ind. ~ Year + offset(Days*) + (1\|ID) | 1,366 | 1,523 | 34.2 | 50.3 | <0.001 |
| Benthopelagic rays | Ind. ~ Year + offset(Days*) + (1\|ID) | 1,146 | 1,320 | 73.7 | 55.7 | <0.001 |
| Guitarfishes | Ind. ~ Year + offset(Days*) + (1\|ID) | 747 | 889 | 64.4 | 200.3 | <0.001 |
| Requiem sharks | Ind. ~ Year + offset(Days*) + (1\|ID) | 949 | 1,115 | 58.8 | 147.0 | <0.001 |
| Hammerhead sharks | Ind. ~ Year + offset(Days*) + (1\|ID) | 665 | 807 | 75.4 | 123.9 | <0.001 |

**log-transformed offset.*

**Appendix S8 - Changes in average species group lengths**Changes in the estimated total length (sharks) and disc width (rays) for the five elasmobranch species groups (sharks in blue, rays in green). Bars indicate the size ranges (size-at-birth to maximum reported size) for the most common species in each species group for comparison, and dark bars indicate the size at maturity range (male/female combined). Points indicate the mean observed size (with error bars indicating standard deviation) of species in the 2021 landing site survey.


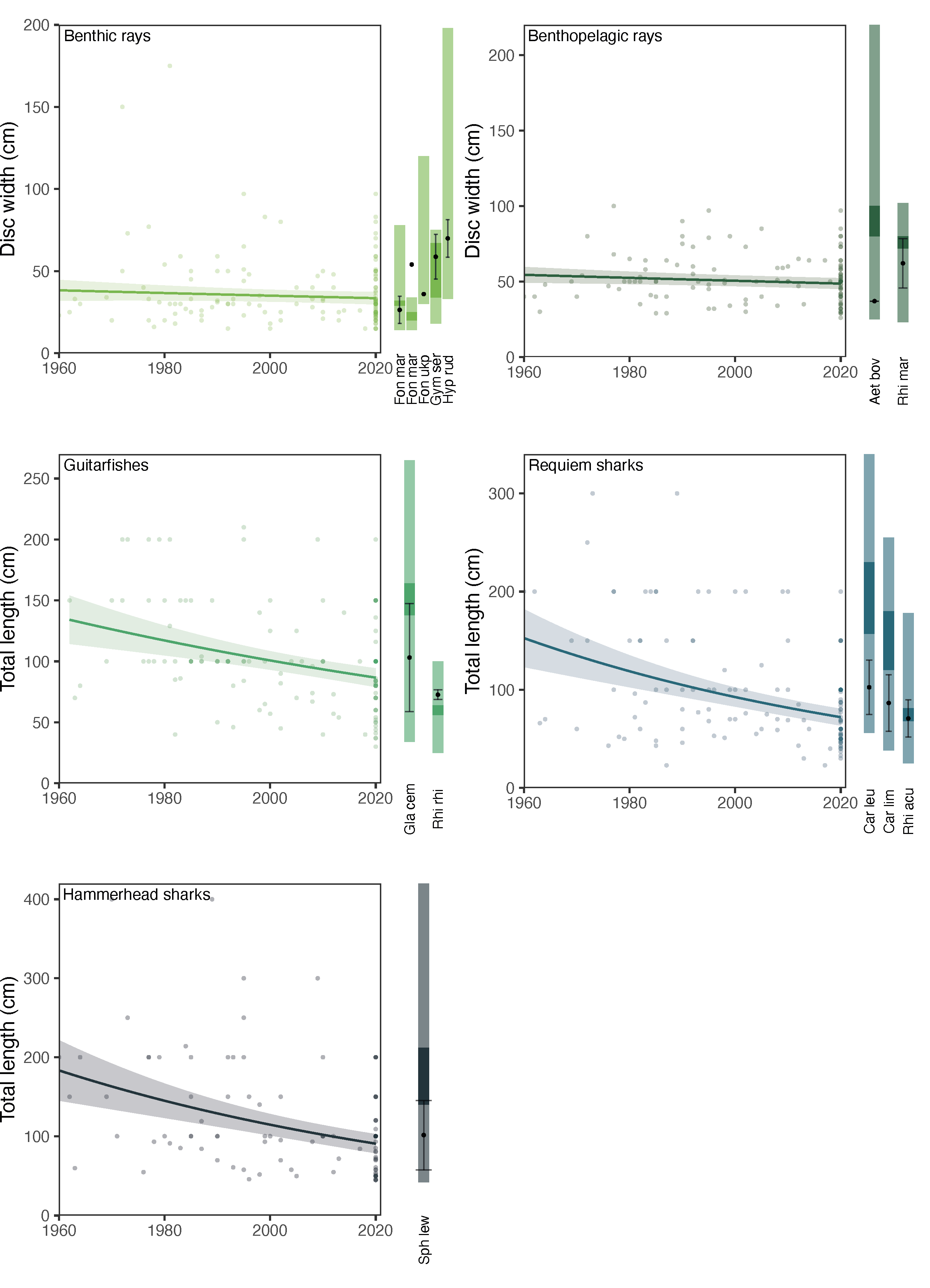

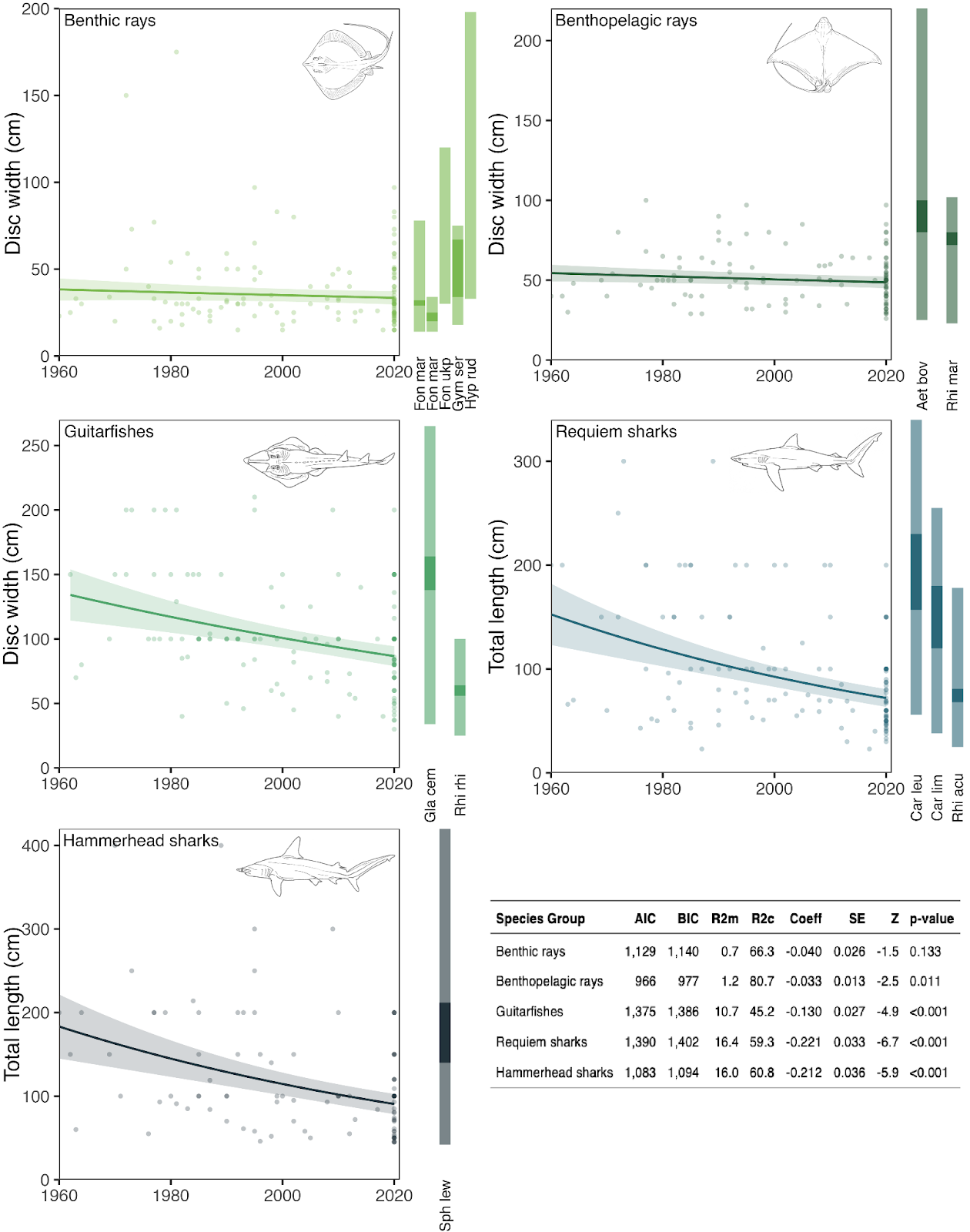


**Appendix S9 - Species group composition**

Changes in species composition with decade-long increments from 1960 to 2020 based on interview responses. Landing site survey (FOP) species composition is also provided but is based on vessels only catching sharks and rays. Ray species groups are indicated in green, and shark species groups in blue. The top three species in the FOP data are shown: *Fontitrygon margarita/margaritella* (FM), *Glaucostegus cemiculus* (GC), and *Rhizoprionodon acutus* (RA).

**
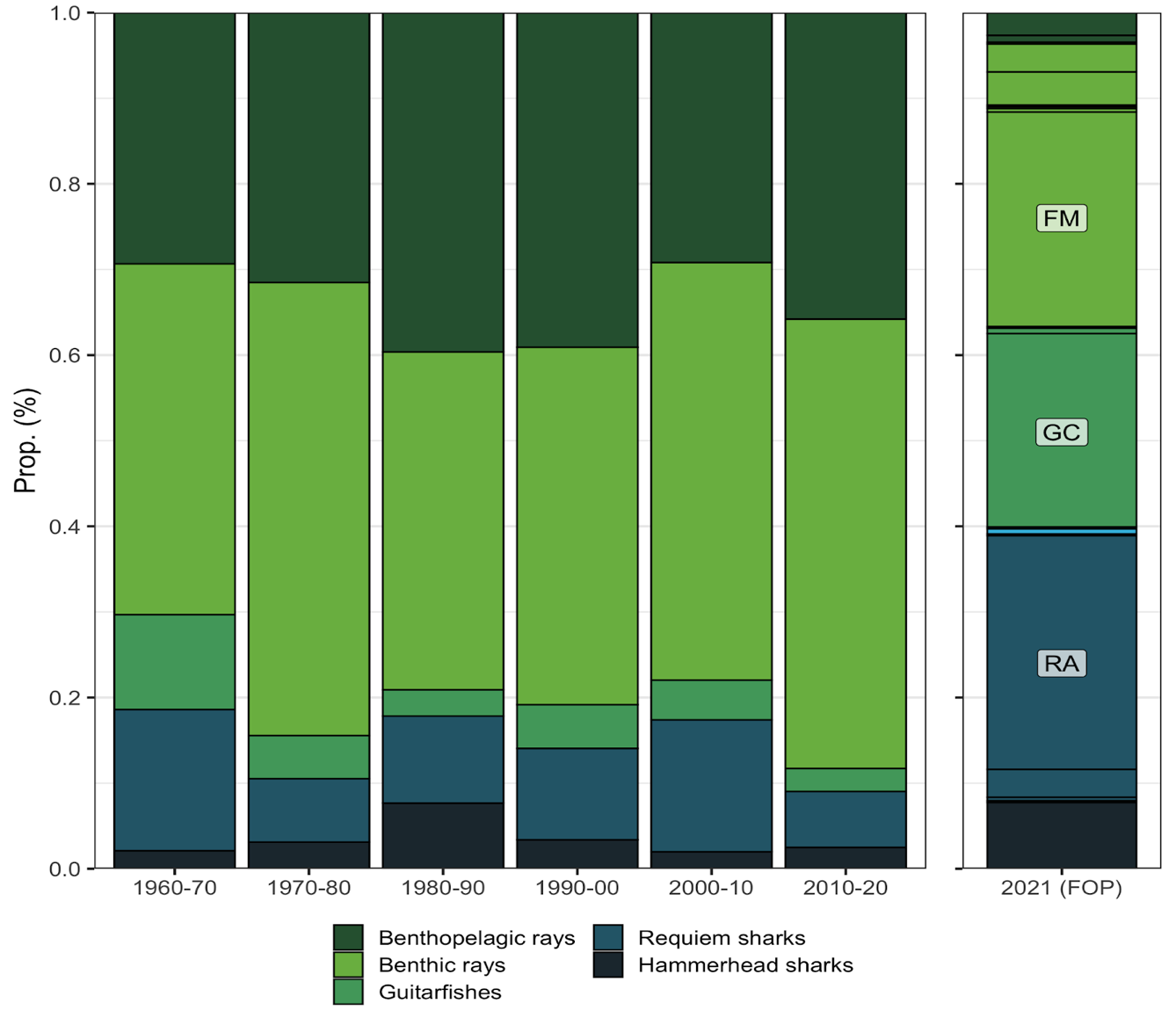
**

**Appendix S10 - Changes in gear abundance, length and soak times.**Generalized linear mixed models to determine changes in the number of gear sets (top), gear length (middle), and gear soak time (bottom) for each gear type: large multifilament nets (dark gray), small multifilament nets (light gray), small monofilament nets (brown), and longlines (turquoise). Points with 95% confidence intervals indicate measurements taken during the landing site survey in 2021, which sampled only fishing vessels catching sharks and rays. The distribution of each model is indicated in the tables with P (Poisson), NB (Negative Binomial), and Gamma.

| 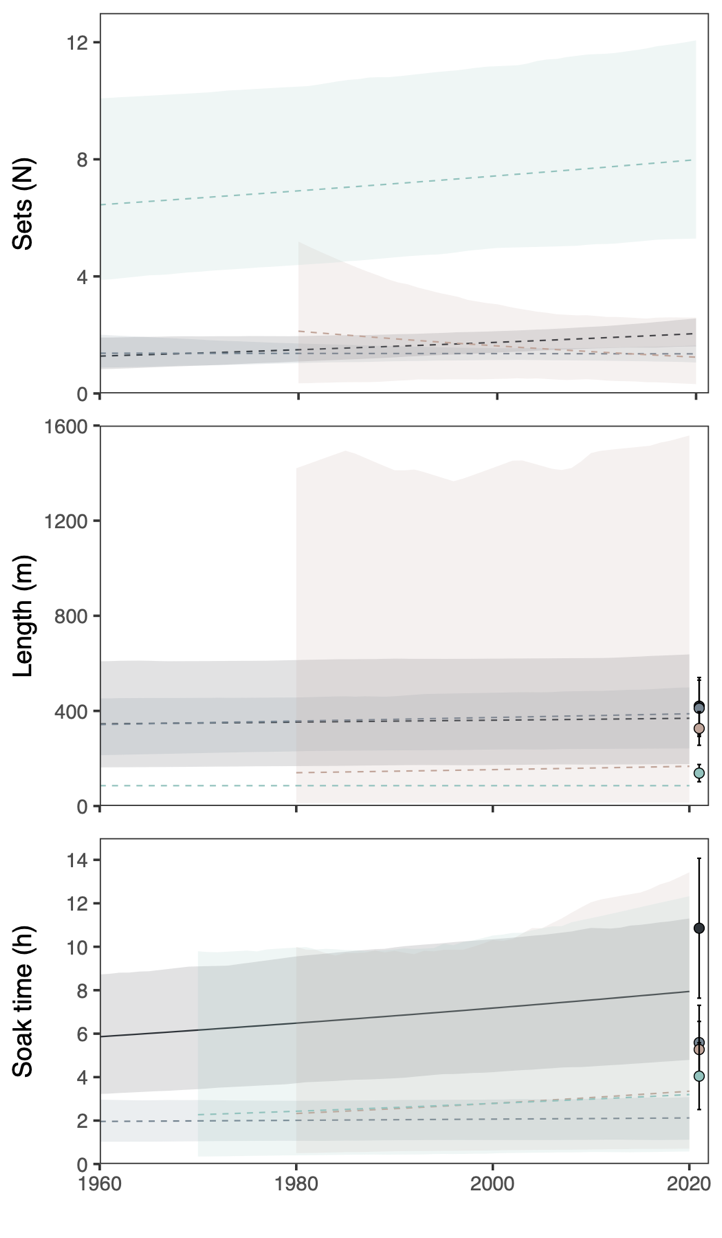 | 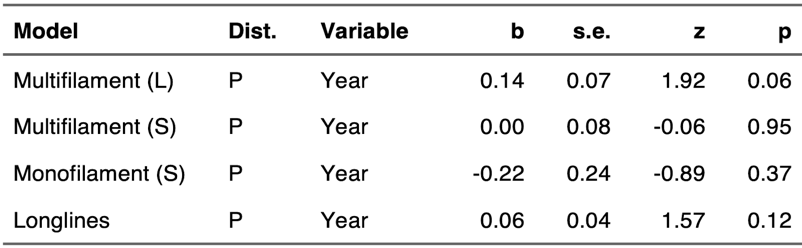 |
| --- | --- |
|  | 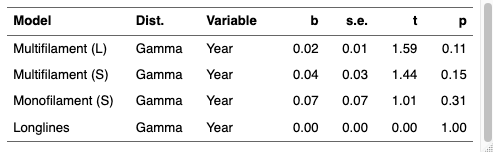 |
|  | 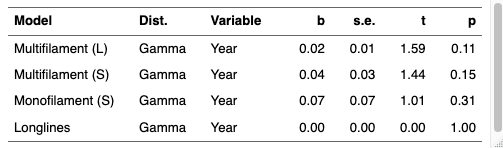 |

**Appendix S11 – Seasonality in Bijagós Small-scale fisheries**

Fishers indicated the number of days fishing per week throughout the year (A). The proportion of respondents that indicated to be active for each month of the year (B). The rain season in Guinea-Bissau is from May to the end of October (blue).

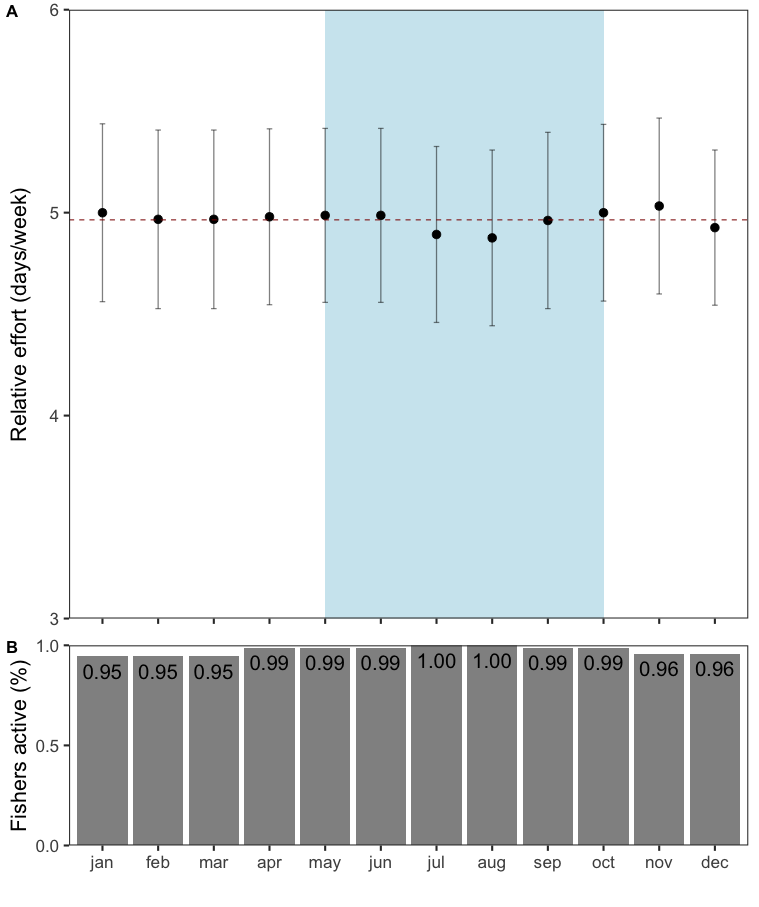

Supplement: Supplementary file 1 — Supporting Information [file COBI-39-e70059-s001.docx]
